# Mathematical Framework for Wearable Devices in the Internet of Things Using Deep Learning

**DOI:** 10.3390/diagnostics12112750

**Published:** 2022-11-10

**Authors:** Olfat M. Mirza, Hana Mujlid, Hariprasath Manoharan, Shitharth Selvarajan, Gautam Srivastava, Muhammad Attique Khan

**Affiliations:** 1 Department of Computer Science, College of Computers and Information Systems, Umm Al-Qura University, Makkah 24381, Saudi Arabia; 2 Department of Computer Engineering, Faculty of Computer Engineering, Taif University, Taif 21974, Saudi Arabia; 3 Department of Electronics and Communication Engineering, Panimalar Engineering College, Poonamallee, Chennai 600123, Tamil Nadu, India; 4 Department of Computer Science, Kebri Dehar University, Kebri Dehar 001, Ethiopia; 5 Department of Mathematics and Computer Science, Brandon University, Brandon, MB R7A 6A9, Canada; 6 Research Center for Interneural Computing, China Medical University, Taichung 406040, Taiwan; 7 Department of Computer Science and Math, Lebanese American University, Beirut 1102, Lebanon; 8 Department of Computer Science, HITEC University, Taxila 47080, Pakistan

**Keywords:** wearable devices, Internet of Things (IoT), deep learning (DL), medical applications

## Abstract

To avoid dire situations, the medical sector must develop various methods for quickly and accurately identifying infections in remote regions. The primary goal of the proposed work is to create a wearable device that uses the Internet of Things (IoT) to carry out several monitoring tasks. To decrease the amount of communication loss as well as the amount of time required to wait before detection and improve detection quality, the designed wearable device is also operated with a multi-objective framework. Additionally, a design method for wearable IoT devices is established, utilizing distinct mathematical approaches to solve these objectives. As a result, the monitored parametric values are saved in a different IoT application platform. Since the proposed study focuses on a multi-objective framework, state design and deep learning (DL) optimization techniques are combined, reducing the complexity of detection in wearable technology. Wearable devices with IoT processes have even been included in current methods. However, a solution cannot be duplicated using mathematical approaches and optimization strategies. Therefore, developed wearable gadgets can be applied to real-time medical applications for fast remote monitoring of an individual. Additionally, the proposed technique is tested in real-time, and an IoT simulation tool is utilized to track the compared experimental results under five different situations. In all of the case studies that were examined, the planned method performs better than the current state-of-the-art methods.

## 1. Introduction

The advancements in medical applications that are increasing for day-to-day life are greatly needed for monitoring groups of individuals at remote locations. Since most users are using updated network information, designing a wireless device embedded within apparel is much simpler. Whenever a monitoring device is designed with the proper apparel, all individuals can carry it to any location, and it is possible to monitor their health conditions at remote locations. Since remote management and monitoring are managed, it is necessary to accumulate all monitored parameters on a cloud platform. Hence, an Internet of Things (IoT) procedure is carried out where different types of infections are examined, and all threshold values are stored on the cloud. Most wearable devices can be incorporated without difficulty in all wireless communication devices, but, in turn, the effects produced by the device for all individuals will increase.

Early diagnosis of any infection needs to be monitored by observing all significant indications that are present in the entire body’s functionality. Whenever wearable devices are designed, as time progresses a higher computational load can be required, as they are related to individual characteristics, whereas, in current-generation wireless networks, microsensor-based chips are used, lacking computational power. As a result of microchips, the efficiency of monitoring devices will increase, and the cost of implementation will be reduced. However, the monitoring features of microsized sensor chips are nearly the same as those of devices with large-scale deployment, though the loss and error functions are reduced.

**Figure 1:**
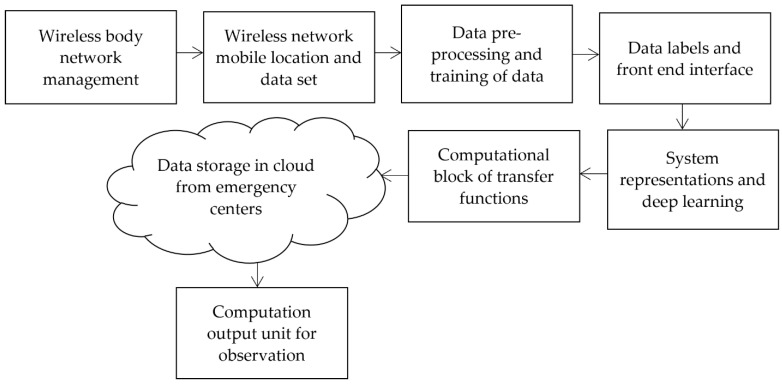
The architecture of the proposed wearable IoT device.

To transform the procedure of monitoring the state conditions and to reduce the errors in identification processes, the proposed method introduces a new state condition that reduces the monitoring time for all types of infections. After monitoring the exact infections using an image processing technique, additional storage features are provided in the proposed method as can be seen in the block diagram of integration shown in Figure 1. From Figure 1, it is observed that wireless devices are managed with modernized technologies. Thus, it is possible to establish a remote monitoring system. Once the device parameters are set, the monitoring location is processed at frequent intervals using the introduced dataset, which contains device and sensor data. Then, the data is pre-processed using image identification parameters and data training is completed at this phase. During the training phase, all devices are labelled, providing a complete interface without errors. Hence, the complete wearable device model is represented with a low error rate, and loss representations are greatly reduced in the system. Finally, data is stored in the cloud, and output units are integrated into the observation process.

### 1.1. Literature Survey

In this section, all pertinent existing models are examined to identify their shortcomings and any potential improvements that could be made to the wearable IoT device by altering a few parametric monitoring systems. Most current models incorporate the same dataset during the observation stage, yet the evaluation results for created systems can be very diverse. Thus, a standard representation form is required, which is given after the survey model. Additionally, as medical applications require a fundamental understanding of all issues, recent research is also reviewed. More details about next-generation networks and their real-time implementation method, which is entirely dependent on network conditions, are given in [1]. According to the implementation plan, to increase the general acceptance rate, it is crucial to assess the growth of products before exposing them to the market. However, one of the disadvantages of next-generation networks is that wearable devices must be synced with all network-related criteria, which is very challenging. Therefore, several applications related to developing body sensor networks that offer basic designs are provided to tackle such synchronization scenarios [2]. Numerous technical specifications are given concerning antenna design in the included designed model, but no separation cases are assessed for various applications. When fog layers are utilized in the identification phases, wearable IoT is incorporated even for detecting the presence of hazardous diseases at an early stage [3]. Fog layers serve as a middleman between all agencies and the cloud, accumulating storage space for individual reports. However, because fog layers demand high bandwidth for transmission and reception, they can also be employed in other identification and diagnosis processes.

Wearable devices must be manufactured following the intended application platform if they are to be implanted in human bodies [4]. To detect the performance of different users, a separate record must be added to the fabrication procedures; as a result, a data record is added to the system. Even though the working model has been fully sensitized, a significant flaw is that not all applications have access to the fabrication conditions in a proper manner. An offloading strategy that boosts the energy of node systems has been developed to address some of the difficulties in fabrication processes [5]. Smart wearable gadgets have a greater capacity for storing significant data amounts and have strong offloading techniques. A distinct platform is generated due to the data storage method, raising the system’s overall operating cost. A wearable gadget was created to detect Alzheimer’s disease in [6]. A large amount of individual storage is needed because the detection method typically involves processing more data. Separate storage spaces cause various applications to be segregated, which makes it much more challenging to process in real-time. Some of the enabling technologies for the Internet of Medical Things (IoMT) have been developed, and serve the entire environment by identifying diseased individuals within specific distance limits [7]. Distance restrictions make delivering a higher data rate for a single data transfer at the transmitter end challenging. A standard technique is required to monitor all required system parameters; even with an increase in data flow, a standard approach is needed.

Even if wireless IoT devices can monitor people’s health, it is still essential to examine the affected person’s allocated space for diagnosis in [8]. An original mathematical model is constructed to test the space allocation, allowing for the distribution of personal space even during times of high demand. Formulations of this kind are used to make critical judgments during times of high risk. The data from wearable devices is also checked using a two-stage paradigm, where accelerometer sensors track all physical activity in [9]. Since minute-to-minute readings are measured as part of this monitoring approach, both the time-varying and invariant parameters are tracked. Even so, both variables produce data with a high density, so the counting procedure is crucial to the processing stage. A posture prediction procedure is carried out using a fusion-based model to avoid high-density data, resulting in error-free data transfer and a significant improvement in posture-prediction accuracy [10]. However, there are a variety of postures that need to be trained using storage techniques. Hence, the training and testing phases call for a collection method involving gathering sensor data. By enhancing the security of each data collection conducted in various physical situations, wearable IoT data transmission accuracy may be increased, as shown in [11]. An automatic monitoring system employing Artificial Intelligence (AI) is introduced as environmental factors change, encouraging cyber–physical systems’ development. Even where a step count strategy is used, it is possible to improve the quality of service for all end users of all wireless IoT devices, by using a defined mathematical approach [12]. 

A fog model is again required to be incorporated in several situations, but, in addition to the procedures above, commonly specified methodologies are represented [13,14,15,16] using an architecture-described system. In the next section, we will discuss a wearable IoT model that uses analytical equations to determine what is wrong with a person within a certain period.

### 1.2. Research Gap and Motivation

All the existing methods, as indicated in Table 1, focus on any one major objective by using medical healthcare services as one of the application platforms. However, if existing methods use wearable devices, then some of the parameters that are needed for time-based monitoring need to be provided. Even existing models need to provide a precise mathematical approach that makes the medical healthcare system function more accurately. However, some of the introduced mathematical methods provide information related to cloud-based services, where the monitored data from medical systems are transmitted, with security features. Further, if a user needs to identify a particular infection at a remote location, additional parameters are required, which is a significant gap in the existing models. Whenever medical healthcare systems are integrated with technology developments using wireless networks, the monitored characteristic features must provide accurate outcomes, and the storage system must function appropriately.

To overcome the gap present in existing models, our proposed system is introduced, where all monitoring is processed only under certain device constraints. A mathematical model is formulated for a device-monitoring system in medical healthcare applications. Even the procedure of device testing is made only by using loop formation provided by a designed mathematical approach. In addition, the proposed method is integrated with a deep learning (DL) algorithm; thus, efficiency at the output unit is augmented with a maximized monitoring distance. Since devices operate at a considerable distance, most of the characteristic features of infections are observed without installing wearable devices in the frame measures. Furthermore, all errors during the transmission process will be minimized; thus, the device functions more accurately.

### 1.3. Contributions

The technique of a wireless monitoring system for healthcare applications, which is designed in the proposed method with wearable devices, is used for solving three major objectives as follows:

- Maximize the monitoring device’s distance, so all infections are identified without connecting to the frame;
- Minimize all errors present in the infection identification and data transmission process, thus increasing the efficiency of the device;
- Integrate a deep learning model for reducing the loss of designed devices with a unique representation of mathematical models.

### 1.4. Paper Organization

The rest of the paper is organized as follows: Section 2 provides a formulation of a system model with the appropriate variables. Section 3 integrates the optimization framework with step-by-step implementation. Section 4 examines the combined outcomes of the proposed method with system formulations, and comparison case studies are also analyzed. Finally, Section 5 concludes with the advantages of the proposed method for future work.

## 2. System Model

This portion, where the designed model is directly incorporated into a real-time setup, formulates the analytical model of wearable IoT devices utilized for recognizing various viruses and infections. Since the classification technique is used to identify people’s ailments, it is crucial to offer resources connected to multiple materials. As a result, the identification procedure is split into two distinct units, each made up of identifiable and unidentified users, where the infirmary centres receive central data. Additionally, wearable IoT devices classify the types of viruses based on blood samples and send the information to local emergency rooms. So, to figure out how far the emergency rooms and hospitals are from each other [5], we can use Equation (1).

(1) $$dist_{i}=max\sum_{i=1}^{n}\frac{A_{i}+P_{i}+t_{i}+h_{i}}{\rho_{i}}$$

In Equation (1), the objective function where the identification distance is maximized. Equation (2) can be used to define the energy representation of local computation tasks when wearable devices with unloading tasks are used [5,21].

(2) $$E_{i}=min\sum_{i=1}^{n}\left(E_{1}+\ldots +E_{i}\right)*\delta_{i}$$

Equation (2) is a reduction function that reduces the workload of each device, because numerous devices are utilized to identify various illnesses, necessitating a greater number of wearables. However, since the device’s data functions are connected by a wireless module, Equation (3) is used to measure the following data waiting time [7,22].

(3) $$Wait_{i}=min\sum_{i=1}^{n}t_{ph}*E_{cm}$$

To illustrate how connected devices work, the third objective function, which is stated as a minimization function, is employed. Equation (4) is used to represent the energy of the communication module [6].

(4) $$E_{cm}=\sum_{i=1}^{n}\frac{p_{cm}}{\beta_{i}}*d_{n}\left(i\right)$$

Equation (4) only applies to situations where people are infected by dangerous viruses; in all other situations, the energy is fully shut off. Equation (5) is used to calculate the sensitivity factor under various ecological conditions [9].

(5) $$S_{i}=min\sum_{i=1}^{n}prob_{i}\left(\frac{H_{i}}{events_{i}}\right)$$

In Equation (5), the number of occurrences shows that control measures using wearable IoT devices, which are built using Equation (6), must be put in place if a person has been infected [12].

(6) $$RI_{i}=\sum_{i=1}^{n}\frac{I_{r}\left(i\right)}{T_{r}\left(i\right)}*100$$

Equation (7) is used for wearable devices to figure out the weighted combination of variables or quality factors since different parameters are used in the representation process [5].

(7) $$Q_{f}\left(i\right)=max\sum_{i=1}^{n}\gamma_{i}*D_{q}\left(i\right)$$

All of the stated Equations (1)–(7), are used to represent the effects of wearable technology and have an optimization mechanism built in to increase operating efficiency. The variables used in the equations are summarized in Table 2. This optimization process is covered in more detail in the next section.

## 3. Optimization Algorithm

It is crucial to introduce an optimization method with efficient mathematical measurements to discover the risk analysis of examined medical photographs. As a result, the described system model is merged with DL which produces beneficial effects by discriminator combinations [23,24]. The main benefit of DL in medical imaging is that it significantly lowers the risk of identification since it makes it easier to identify more infectious diseases using a set of implicit functions. Additionally, it is crucial to create a feature automation procedure for diagnosis, so that a stated logical structure can be implemented without causing any side effects. Additionally, generative measurements have a vastly improved complexity of conjunction for supplying matching features compared to standard measurement types. Therefore, even for big dataset models where identification time is minimized, a fixed distribution set is used in the identification processes. However, as shown in Equation (1), the data distribution model utilizing DL can be controlled using training samples, as indicated in Equation (8) [21].

(8) $$train_{s}\left(i\right)=min\sum_{i=1}^{n}prob_{g}\left(i\right)+prob_{d}\left(i\right)$$

Equation (8) shows that the probability function of DL is a minimization problem, where the wearable object spreads images it has collected. Since it is possible to find loss functions during these transmissions, it is essential to use Equation (9) to minimize data loss [22].

(9) $$loss_{i}=min\sum_{i=1}^{n}l_{s}\left(i\right)+l_{p}\left(i\right)$$

To reconstruct the target system using several wearable items, the loss periods must be kept to a minimum. So, Equation (10) is used to measure the signal matrix of wearable devices [21,22].

(10) $$O_{i}=\sum_{i=1}^{n}T_{o}\left(i\right)+\left[\begin{array}{cc} S_{1} & S_{2}\\ S_{i} & S_{n} \end{array}\right]$$

**Figure 2:**
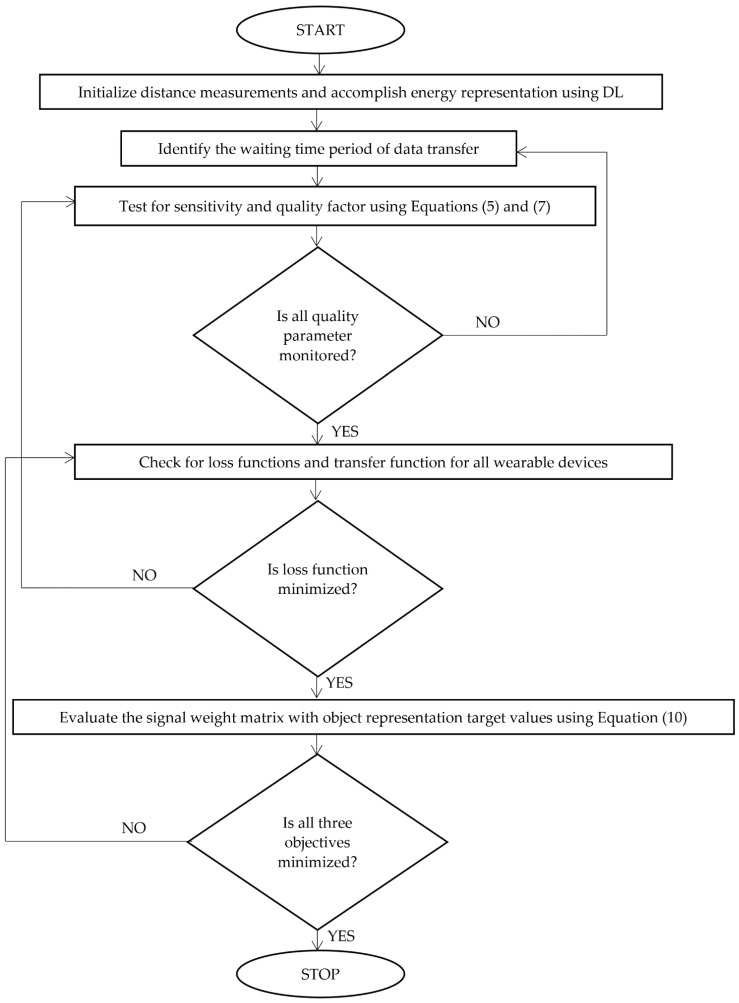
Flowchart of DL for wearable healthcare applications.

Equation (10) states that the signal representation matrix for loss functions must be reduced to a minimum of 0.01%, making it possible for wearable devices to communicate without interruption, even at greater separation distances. Figure 2 shows the integration method’s careful, step-by-step application utilizing DL.

### Step-by-Step Implementation of DL Using Adversarial Networks

*Input:* Initialize the maximum distance in terms of identified area, type of patients, periods, and demand for capturing images in wearable devices using the representative values of the matrix functions $A_{i}\;\left(A_{i}\leq i\leq n\right)$, $P_{i\;}\left(P\leq i\leq n\right)$ and the time matrix $t_{i}$;

*Output:* Minimize the load and waiting time and maximize the distance and quality of service factors:

Step 1: The objective function is constructed with different types of patients’ data and one demand persistent value using $\rho_{i}$;

Step 2: Establish the relationship between the different energy representations and working functionalities of devices that must be followed by waiting periods $Wait_{i}$ with 1≤i≤n, and its wireless module representation values $E_{cm}$ at different marginal functions;

Step 3: while $\left(dist_{i}<N\right)$ do.

- Select the captured images and measure the communication module energy representation values using different data sets in a systematic way for computing the throughput, by using Equation (3);
- Verify the value of $S_{i}$ and $E_{cm}$ using the probability value set;
- If the complexities of identification are higher, $S_{i}$ is not at $\left(S_{i}<N\right)$ do;
- Divide the probability of different events using the number of affected users $H_{i}$ and $events_{i}$, which ensures different ecological conditions using Equation (5) $H_{i}$ with 1≤i≤N into *N* number of affected points;
  //Training sample phase
- Update the infected region and total region areas using a spreading ratio matrix with minimized loss function using sample and prediction loss as shown in Equation (9);
  //Loss phase
- Select the number of loss functions with wearable signal representation values of different training samples with separate image analyses in a single output $train_{s}\left(i\right)$, as defined in Equation (8);
- Update the object representation values of the relative positions and identified position, followed by measurement of the data quality index, and compute the relative weight of all parameters $\gamma_{i}$, as defined in Equation (7);
- Identified infections at each point are updated by using the probability of the generator and data that is designed for the image signals;

$$N_{i}\left(new\right)=N_{i}\left(old\right)+1;$$

End;

Step 4: If $\left(loss_{i}<N_{i}\right)$ then $loss_{i}\leftarrow 0$; //Interchange the existing solution in the current loop with the new solution; End if;

Step 5: If $\left(dist_{i}\;\left[0,\;1\right]<loss_{i}\right)$ then Re-initialize the images that are taken with the new processing technique; Obtain the overall best solution; End if;

Step 6: If $\left(Q_{f}\left(i\right)>N\right)$ //Existing solution is replaced with the new solution

$$N_{new}=N_{i};$$

$$N_{old}=N_{i};\;$$

//Attain the most feasible solutions for determining the overall best solution; Increment the count $N_{i}$ by 1; Return the best overall solution; End.

## 4. Experimental Outcomes

For wearable IoT devices, the integrated process of a defined system model and optimization algorithm must be tested in real-time using carefully defined experimental models. To identify various infections, real-time verification with a simulation setup is used, and the parametric values that help enhance the effectiveness of the suggested system are offered. All conditions are detected in all developed device models, which is the main reason for merely supplying an experimental value. However, it is crucial to identify which wearable device performs more effectively than the other devices in various parametric values. Additionally, the entire dataset is provided in the input layer of the suggested methodology. This is only gathered from multiple treatment facilities because it is much more accurate than other data set representations. Further, a real-time application is created, where all defects in the application segments are fixed after extensive testing and simulation over more than 1000 phases.

Additionally, the proposed technique incorporates extra data based on the availability of beds at all surrounding hospitals during times of emergency. As a result, if any infections are seriously impacted, the wearable device will be able to detect them and, using location data, alert local treatment facilities. In real-time, the visualization process of wearable devices is carried out by examining the analytical framework under five different scenarios, as follows:

Scenario 1: Measurement of distance;

Scenario 2: Waiting periods;

Scenario 3: Sensitivity factors;

Scenario 4: Quality of measurement;

Scenario 5: Loss periods.

All the input parameters used for designing a particular wearable device are provided in Table 3. The primary application of the proposed wireless wearable device, which operates with a DL algorithm, is to observe the type of infection imposed on an individual. In current changing environments, most people must undergo regular medical checkups to safeguard themselves from different types of infected viruses in the system. However, due to the lack of remote monitoring systems, it is possible to continuously monitor all the infections quickly, so that the lives of surrounding people can also be saved. Hence, the proposed method is applied in medical applications to discover all infected viruses in an anthropological frame. All the values represented in the simulation tables are achieved only after careful experimentation and testing of wearable devices. In addition, all defined values denote the device’s performance measures that operate under proper environmental conditions. The representation values are simulated using the MATLAB IoT toolbox for better empathetic constraints. Thus, all parameters, including the sensitivity of the designed system, are analyzed. However, to provide a real-time representation comparison, the form of the figures is also simulated in MATLAB using a 3-dimensional (3D) plot. The simulated plots indicate that the proposed method performs much better than existing techniques regarding various parameters, where all affected figures are described using loop-based codes. For simulating corresponding representations, a hardware processing unit is directly connected to MATLAB using a serial cable port. Therefore, the plot is simulated in MATLAB with a DL integration process.

All primary scenarios are integrated into IoT-based hardware and software platforms using an MIT application intervention. Still, all output units are directly connected with the MATLAB IoT processing tool, as simulation characteristics with a comparison state need to be analyzed. In the application platform, supporting blocks for wearable IoT devices are created and connected with the user interface; thus, the application layer’s functionalities are introduced. During this type of functionality implementation, all properties of wearable devices are added at the initial phase of application creation using proper list-view selections. The outcomes are presented for all observed periods since all parametric values cannot be measured if findings are evaluated in subsequent weeks. Additionally, the study results offer potential low-cost ways to improve the physiological health of all patients. The following are comprehensive descriptions of each scenario.

### 4.1. Scenario 1

**Figure 3:**
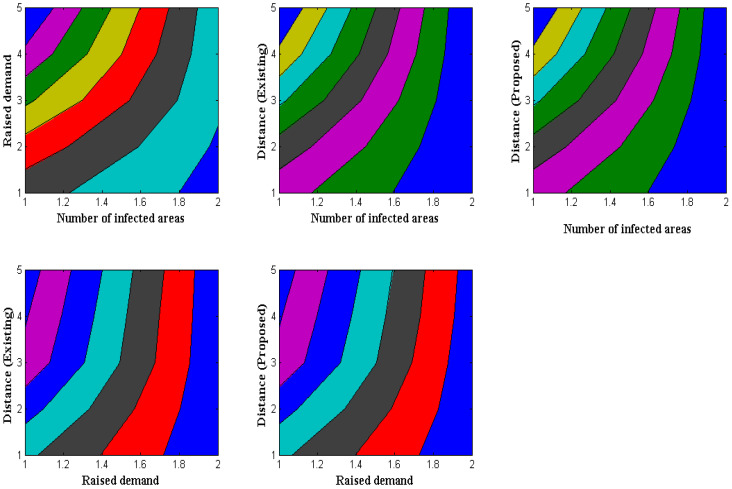
Distance and demand measurements.

Wearable-device distance measurement is crucial because some people will keep their devices close to their bodies if they are particularly sensitive to certain membrane regions. Therefore, it is crucial to gauge the distance at which all users are permitted to remove their wearable technology and store it. The proposed method clarifies distance by looking at the afflicted areas, where certain illnesses can be detected in specific locations. In the observed technique, identification with information measurement time is also considered a summation value. Another unique feature of the suggested method is that total values are separated by measuring the distance of demand in a specific area. Furthermore, fitted wearable devices are not physically harmed while measuring distance, conforming to a standard pattern for distance measurements. After separating all new values by demand, Figure 3 shows a simulation of distance measurement.

Figure 3 and Table 4 show that a comparison is made with the existing approach, which offers the least amount of distance measurement compared to the suggested model. In this example study, there are 120, 180, 260, 340, and 400 contaminated regions, respectively. Since only dataset-1′s data contains the aforementioned afflicted locations, low measurement results are used. It is crucial to give the required quantity of resources to specific locations in afflicted areas, where the total resource demands are 32, 44, 57, 61, and 65, respectively. The total distance of separation in the case of the suggested method is maximized to 1.6, 2.8, 3.4, 4.9, and 5.7 m, due to the separation of the demand factor. In contrast, the current method minimizes it to 1.3, 1.7, 2, 2.2, and 2.5 m, making it impossible to allocate even the demand factor. In the proposed technique, where all demand resources can be assigned, the distance of separation is, therefore, optimized through demand-factor measurement.

### 4.2. Scenario 2

**Figure 4:**
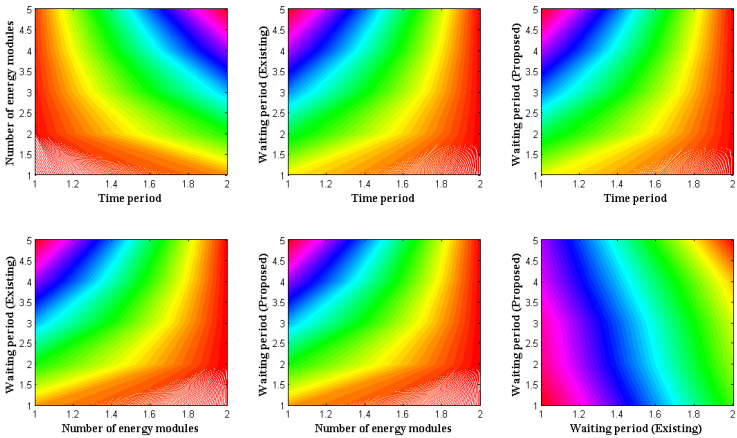
Waiting periods at high energy.

Wearable technology that transmits data must offer a low energy value for sending a single data packet to a destination. As a result, the rate of energy measurement is seen for various wearable devices, where it must be reduced while maintaining functionality in specified devices. The waiting time for all data will be minimized, using the minimization objective function. A wireless communication module was introduced for this sort of reduction target, significantly reducing data transfer time. The communication module can also be created using a low-cost design that considers the lifespan of all required components. As a result, the wireless module’s reproduction rate and period will give precise results linked to the waiting period for the data that are transmitted by each user end. The simulation results of waiting times for various wearable devices are shown in Figure 4.

Figure 4 and Table 5 show that the periods of the devices vary from 10, 20, 30, 40, and 50 s, with the number of energy modules being represented for each period in steps of 100. The devices’ waiting times are calculated after the reproduction rate is measured, to ensure the least amount of waiting time possible. In the comparative state, the existing approach from [3] offers a single data segment with a higher waiting time than the suggested way. This may be tested for 30 s using 500 energy modules, where the total time spent in the unload condition for the proposed technique is equivalent to 1.1 s. However, with the identical setup, the current approach only delivers 2.17 s, so an increase in waiting time is noted. Inadequate data proportions exist in the present method due to increased waiting time. However, this problem is resolved in the proposed method by employing adversarial tactics.

### 4.3. Scenario 3

**Figure 5:**
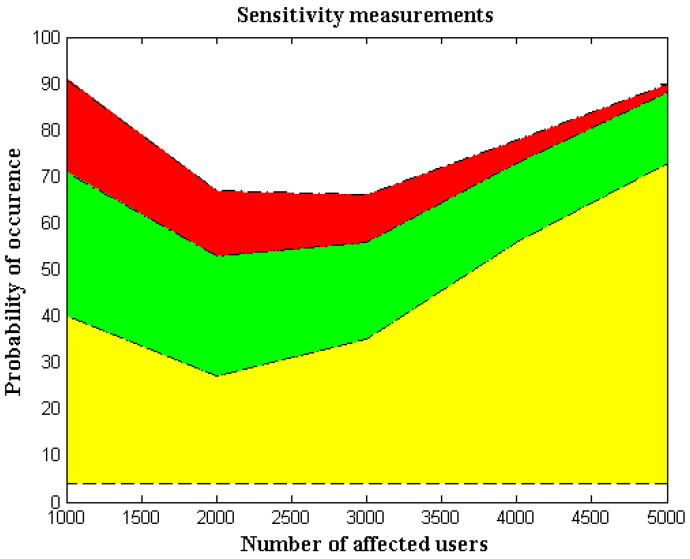
Measurements of sensitivity.

In this case, probability analysis estimates the sensitivity parameter by counting the number of incidents. The number of infected people is directly separated by utilizing event-measurement cases for sensitivity measurements in wearable IoT devices. Reduced device sensitivity is crucial as a safety precaution, because wearable technology may harm all users if the sensitivity factor is higher. Additionally, since direct radiation will be present across the entire network, it is necessary to eliminate all direct radiation from the system-generating process so that sensors subject to highly sensitive conditions will remain intact. Additionally, since the probability of measurement alterations is significantly larger when an entire network is infected, the system must be modified to eliminate mobile devices’ sensing capabilities. Even though the probability analysis model uses separation techniques, it is still possible to regulate the full spread ratio by looking at all the infected areas. Figure 5 shows the comparison scenario and simulation results for the sensitivity parameters.

Figure 5 and Table 6 make it clear that the number of impacted users is far higher, so only a small number of affected cases—1000, 2000, 3000, and 5000—are taken into account. For each affected user, the probability of occurrence is computed as 40, 27, 35.56, and 73, respectively. Since both infected and total regions are observed for each probability of occurrence, individual probability values are minimized. Furthermore, it makes sense in a comparison situation that the existing method’s sensitivity percentage is substantially higher than our proposed method’s. This may be demonstrated by using the 5000 affected users and a high repeated probability of 73; in this scenario, the proposed method’s sensitivity is just two percentage points, but the proposed method’s sensitivity is 15 percentage points. The cost of measurements goes up, since the existing process, which is less sensitive than the suggested method, requires more control measures.

### 4.4. Scenario 4

Every developed communication equipment used in various applications must offer high-quality service to every network user. Similar to how monitoring needs to be improved, intended wearable IoT devices must provide high-quality service expressed in terms of data. Therefore, a mathematical model is created to enhance the service quality by considering the relative importance of all the system factors. The relative weights from Table 7 are significantly different from the present procedure because, in earlier circumstances, only data weights were considered without any specified formulations. The total reproduction rate improves the service because it measures the system’s relative weight and the data quality index at the transmitter end.

**Figure 6:**
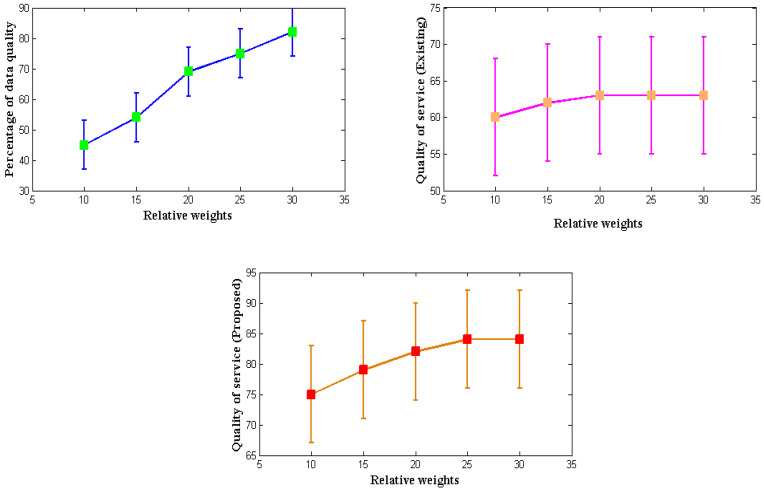
Quality of service measurements.

Even though it is possible to measure service quality using a variety of control measures, the data quality index for wearable devices will decrease if controls are implemented; so, in the proposed method, controls are provided in the form of a relative weight factor. The simulated result of service quality is shown in Figure 6. It is reasonable to assume that the proposed method will provide a much higher service quality than the current approach, based on Figure 5 and Table 7 [3]. To simulate the quality-of-service measurement, weights of 10, 15, 20, 25, and 30 g are considered. These weights include microsensors made of light materials. The percentage of data quality provided during such an operation is 45, 54, 69, 75, and 82, respectively, and the rates above are replicated in the quality-of-service factor. When the reproduction rate is measured and compared to the existing method, the latter yields a service rate of 84% for high relative weights and data quality, compared to 63% for the exact requirement for consistent quality of service as the relative weights rise. Low parametric determinations also cause the low quality of the index given by the current method. However, the proposed method can improve the quality of service for high measurement values.

### 4.5. Scenario 5

**Figure 7:**
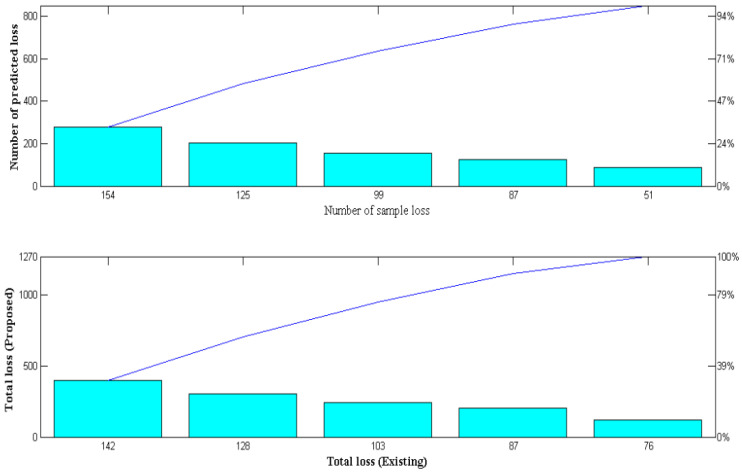
Comparison of loss factor.

In this scenario, the loss of measurement in wearable IoT devices is measured and handled as significant parametric real-time measurements. After the detection process and measurements are taken to reduce the amount of duplicate data in the system, it is crucial to understand the amount of data transmitted. All the detected data must be represented in the output system to prevent the loss of the input data measurements since wearable devices are used in medical diagnosis. However, real-time data loss will occur; as a result, adversarial deep learning optimization is included in the proposed method to detect the sample and prediction loss periods. However, training samples of the generator system must be measured precisely, where inputs given in various arrangements will be processed in a standard mode to detect such losses in the system. The proposed system’s data loss is significantly reduced due to common mode representation, and its simulation results are shown in Figure 7. 

Figure 7 and Table 8 show that there were 89, 124, 153, 205, and 279 data sample losses for the input representations, respectively. The designed system predicts that the following minimum losses will occur for each sample loss: 51, 87, 99, 125, and 154, respectively. If the total loss is less than the expected loss, then the wireless IoT device performed well in the data transmission cases. The total data loss of the proposed method is lower when compared to the existing method [3], but good performance is only attained at high sample data representations. This can be demonstrated by a sample loss of 279 and a predicted loss of 154, where the overall data loss is 142. Thus, it is possible to minimize data loss and increase system throughput using deep adversarial learning. Even if a lot of data are given, the total loss can still be less than expected [19,20].

### 4.6. Performance Analysis

The performance analysis determines whether the designed devices function appropriately with unique characteristic features compared to other existing methods. Therefore, in this section, the robustness characteristics of wearable devices are examined, and comparisons with existing models are also provided.

### 4.7. Robustness Characteristics

**Figure 8:**
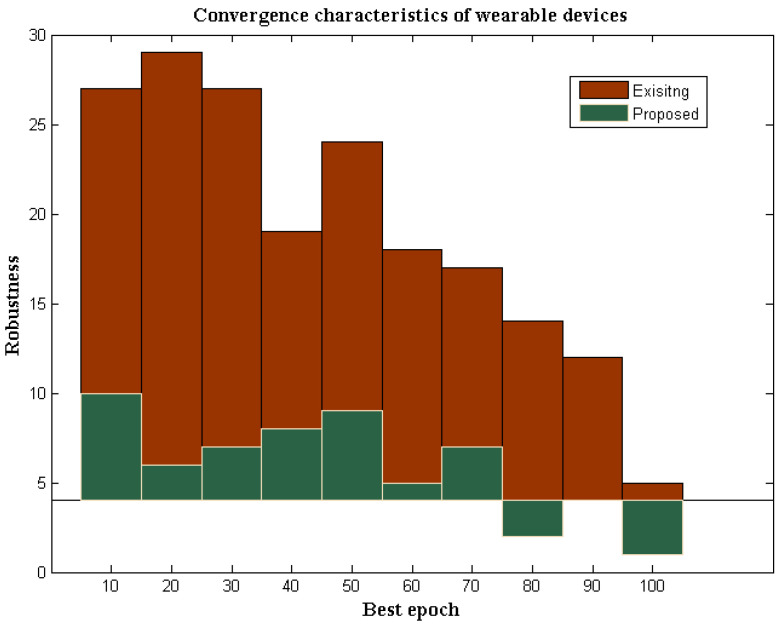
Robustness characteristics.

A wearable device that operates in IoT must be effective under all circumstances, with low robust conditions. However, wearable devices are subject to 4% of robustness if operated in a hostile environment, whereas in standard environmental cases, 1% of robustness will be present [4]. The robustness of wearable devices with DL determines the strength of identification when the data are transmitted in a particular system. If both transmission and identification strengths are much higher, then high efficiency at output units can be achieved. In addition, in wireless devices with communication media, the most potent transmitters are not used; hence, the strength of identification is much lesser. However, in the proposed method using deep learning, better optimization characteristics are achieved. Therefore, the identification process is made much simpler. The robustness characteristics of the proposed and existing method are provided in Figure 8.

From Figure 8, it can be observed that the strength of the proposed wearable devices for identifying a particular infection is much higher compared to the existing method. To verify the robustness characteristics, more iterations are considered from 10 to 100, where, during each variation, the strength of identification changes between lower and upper values. However, during these changes, the proposed method maintains tolerable limits, as the threshold capacity of the device is predefined in the wearable device. However, in the existing method [3], even if the low values are changed, tolerable limits are not maintained in the system. As a result of low tolerable conditions, the robustness of the existing method remained at 5%, while the proposed method provides 1% robustness to the maximum extent. These comparison values are plotted concerning the simulation time standard in MATLAB, but the proposed wearable devices provide much low robustness in real-time, as the loss factor is minimized.

## 5. Conclusions

Wearable Internet of Things (IoT) devices not directly inserted into any human body system are used to examine the most crucial need in the current generation of networks used for identifying various infections. However, because a communication unit is present, all central servers in the system can be directly contacted by wearable devices, allowing all specialists to provide medical information remotely. The design of wearable devices must be distinctive, to identify every infection at early onset, where possible. For this reason, mathematical design parameters are introduced in the proposed method to design a wearable device capable of identifying parametric values. Additionally, with the designed formulations, it is crucial to incorporate an optimization algorithm that offers successful solutions under low-risk circumstances. As a result, a deep learning procedure is included in our model. Thanks to the integrations above, all offloading requirements must be met to convert standard devices to smart devices. In addition, a cloud-based data-collection unit is present, where IoT device outputs are sent for decision-making. The proposed method’s results are validated using five experimental scenarios, including distance measurements, data waiting times, sensitivity levels, quality assessments, and loss assessments. The proposed method offers the best results, by about 68 percentage points more than the existing state-of-the-art method, according to the parametric output values for all the test case verifications of designed wearable devices. By making the materials used lighter, wearable device models with advanced designs can be possible in the future, and automated optimization processes can be used to improve security and privacy in alternate ways.

## Figures and Tables

**Table 1:** Background and objectives (existing vs. proposed).

References	Background	Objectives
[16]	Overview of medical devices and application platform for wearable devices	Minimize the cost of implementation
[17]	Detection of interruptions that are present in monitoring systems for wireless devices	Minimize the number of IoT data interruption
[18]	Possible developments in wireless network applications for medical healthcare	Minimization of cost
[19]	Fabrication design of wearable devices for different applications	Minimization of congestion
[20]	Design of wideband antennas for wireless communication transfer	Maximization of coverage
Proposed	Deep learning approach for wearable devices	Multi-objective framework with minimization of loss, energy and errors

**Table 2:** Mathematical notations.

Variables	Description
$A_{i}$	Identified area of infection
$P_{i}$	Different types of patients with infections
$t_{i}$, $h_{i}$	Time period and infirmaries of identification
$\rho_{i}$	Demand for persistence in a particular area
$E_{1}+\ldots +E_{i}$	Energies of different wearable devices
$\delta_{i}$	Work functionality of devices
$t_{ph}$	Time period of mobile-connecting devices
$E_{cm}$	Energy of communication module
$p_{cm}$	Power delivered to the communication module
$\beta_{i}$	Throughput of the device module
$d_{n}$	Size of data to be transmitted
$H_{i}$	Group of affected users
$events_{i}$	Occurrence of different events
$I_{r}$, $T_{r}$	Individuals in infected regions and total covered regions
$\gamma_{i}$	Relative weights of all parameters
$D_{q}$	Data quality index
$prob_{g}$, $prob_{d}$	Probability of generator and data
$l_{s}$, $l_{p}$	Sample loss and prediction loss periods
$T_{o}$	Object representation target
$S_{1}..S_{i}\ldots S_{n}$	Wearable signal-representation matrix

**Table 3:** Input specifications (existing vs. proposed).

Key Features	Existing [3]	Proposed
Package size	4 × 5.2 × 1.3	2 × 2 × 0.7
Sensor power	High power greater than 5 volts	Ultra-low power with a three-axis accelerometer
Noise density	50	22
Current consumption	0.89 mA	0.55 mA
Maximum distance	2.5 m	5.7 m
Sensitivity	15	2
Gain bandwidth	4 kHz	8 kHz
Memory unit	100 GHz	500 GHz
Run mode	12 microamperes	30 microamperes

**Table 4:** Raised demand and distance.

Number of Infected Areas	Demand	Distance [3]	Distance (Proposed)
120	32	1.3	1.6
180	44	1.7	2.8
260	57	2	3.4
340	61	2.2	4.9
400	65	2.5	5.7

**Table 5:** Energy modules with waiting periods.

Period	Number of Energy Modules	Waiting Period [3]	Waiting Period (Proposed)
10	100	2.33	1.25
20	300	2.21	1.16
30	500	2.17	1.1
40	700	2.06	1
50	900	2	0.8

**Table 6:** Percentage of sensitivity.

Number of Affected Users	Probability of Occurrence	Percentage of Sensitivity [3]	Percentage of Sensitivity (Proposed)
1000	40	31	20
2000	27	26	14
3000	35	21	10
4000	56	17	5
5000	73	15	2

**Table 7:** Quality of service with a relative weighting factor.

Relative Weights	Percentage of Data Quality	Quality of Service [3]	Quality of Service (Proposed)
10	45	60	75
15	54	62	79
20	69	63	82
25	75	63	84
30	82	63	84

**Table 8:** Loss measurement values.

Number of Sample Loss	Number of Predicted Loss	Total Loss [3]	Total Loss (Proposed)
89	51	117	76
124	87	203	87
153	99	245	103
205	125	305	128
279	154	400	142

## Data Availability

The data presented in this study are available on request from the corresponding author. The data are not publicly available due to the program code transfer path.
